# Clinical and biochemical profile in adolescent and adult polycystic ovary syndrome patients

**DOI:** 10.12669/pjms.41.9.12228

**Published:** 2025-09

**Authors:** Bakht Babar, Ghulam Farooq, Misbah Manzoor, Hashim Khan

**Affiliations:** 1 Bakht Babar, FCPS Medicine, FCPS Endocrinology, Assistant Professor Endocrinology Department of Diabetes, Endocrinology and Metabolic Diseases, MTI Hayatabad Medical Complex, Peshawar, Pakistan; 2 Ghulam Farooq, FCPS Medicine, PGR Endocrinology Department of Diabetes, Endocrinology and Metabolic Diseases, MTI Hayatabad Medical Complex, Peshawar, Pakistan; 3 Misbah Manzoor, FCPS Cardiology, Registrar Cardiology, Department of Cardiology, MTI Hayatabad Medical Complex, Peshawar, Pakistan; 4 Hashim Khan, MBBS, House Officer, Endocrinology Department of Diabetes, Endocrinology and Metabolic Diseases, MTI Hayatabad Medical Complex, Peshawar, Pakistan

**Keywords:** Adolescent, Adult, Polycystic Ovary Syndrome, Insulin Resistance

## Abstract

**Objective:**

To compare the clinical and biochemical profiles of adolescent and adult patients with Polycystic Ovary Syndrome (PCOS) and to evaluate the impact of insulin resistance on metabolic parameters.

**Methodology:**

A cross-sectional study was conducted at a tertiary care hospital from June to December 2024. A total of 150 PCOS patients, diagnosed using the Rotterdam criteria, were included. Clinical features (BMI, menstrual irregularities, hirsutism and family history) and biochemical parameters (fasting glucose, insulin, HOMA-IR, lipid profile and hormone levels) were analyzed. Patients were stratified into insulin-resistant and non-insulin-resistant groups for comparison. Insulin resistance was defined using HOMA-IR cut off value of more than 2.5.

**Results:**

Adolescents had lower BMI (25.5 ± 3.8 vs. 29.1 ± 4.9 kg/m², p < 0.001) and more frequent menstrual irregularities (90.7% vs. 49.3%, p < 0.001) than adults. Insulin resistance was observed in 85 (56.7%) patients and was associated with significantly higher fasting insulin (18.2 ± 4.8 vs. 9.8 ± 2.9 μU/mL, p < 0.001), HOMA-IR (4.8 ± 1.2 vs. 2.3 ± 0.6, p < 0.001) and higher LDL with lower HDL levels.

**Conclusions:**

Significant age-related differences in clinical and biochemical profiles exist among PCOS patients, along with insulin resistance status. Early identification of metabolic risks is crucial for timely intervention.

## INTRODUCTION

Polycystic ovary syndrome (PCOS) is one of the most common endocrine disorders in women of reproductive age, with a global prevalence ranging between 6% to 21%.^1^ This syndrome adversely affects female fertility and raises the risk of cardiovascular illnesses, diabetes, dyslipidemia, obesity and psychological disorders.^2^ In 2017, 1.55 million women in fertile age groups were diagnosed with PCOS, of which, 17.23% were women aged 21-30 years old.^3^ Menstrual abnormalities, polycystic ovarian morphology and hyperandrogenism are all hallmarks of this diverse disease.^4^,^5^ The Rotterdam criteria remain the mainstay for the diagnosis of PCOS.^6^ However, diagnosing adolescents with PCOS often presents with a unique challenge due to the overlap between physiological adolescent menstrual irregularities and pathological ovarian dysfunction. Adolescents often exhibit transient features of PCOS, such as irregular cycles and multifollicular ovaries, which may normalize with time.^7^

The clinical presentation of PCOS varies widely among different individuals and age groups. Obesity is a particularly prevalent comorbidity in PCOS that can exacerbate insulin resistance, contributing to the worsening of metabolic and reproductive dysfunction.^8^ However, even lean PCOS patients exhibit metabolic disturbances, highlighting the complex pathophysiology of the disorder. Adolescents present with distinct metabolic abnormalities that may differ from adults, necessitating age-specific diagnostic and management approaches.^9^

Biochemically, PCOS encompasses increased serum androgen levels (testosterone androstenedione and dehydroepiandrosterone sulfate), an elevated ratio of luteinizing hormone (LH) to follicle-stimulating hormone (FSH), as well as insulin resistance indicators such fasting insulin and the homeostasis model assessment of insulin resistance (HOMA-IR). Hyperinsulinemia, a key pathophysiological driver in PCOS, which amplifies ovarian androgen production and disrupts folliculogenesis.^10^ Understanding the age-related variations in clinical and biochemical profiles is crucial for optimizing care in PCOS patients. Early diagnosis in adolescents allows for timely lifestyle interventions and targeted medical therapies to delay the development of chronic diseases, including heart disease, metabolic syndrome and infertility. Moreover, individualized treatment approaches based on biochemical findings can enhance therapeutic outcomes.

In this study, we assessed the biochemical and clinical characteristics of polycystic ovary syndrome (PCOS) in adolescents and adults to delineate age-specific differences that could refine diagnostic and therapeutic approaches. In Pakistan very limited data regarding this difference in clinical and biochemical parameter of adult and adolescent PCOS patient is available. By analyzing key metabolic and hormonal parameters, we will not only be able to fill this gap but we also aim to add to the expanding library of PCOS-related literature and provide evidence for personalized treatment strategies across different age groups.

## METHODOLOGY

This six-month cross-sectional study was performed at a Hayatabad Medical Complex, Peshawar from 20^th^ June to December 2024.using non-probability consecutive sampling method. Informed consent was secured from all participants.

### Ethical Approval:

It was obtained from from Ethical review committee (Approval number 1820, Dated 20^th^ June, 2024)

A total of 150 patients met the inclusion criteria for polycystic ovary syndrome (PCOS) according to the Rotterdam criteria. In order to diagnose polycystic ovary syndrome (PCOS), the Rotterdam criteria state that two out of three of the following must be present: (1) Oligo- or anovulation (menstrual cycles that last more than 35 days or less than eight cycles per year). (2) Clinical or biochemical hyperandrogenism (elevated serum testosterone or clinical signs such as hirsutism with a Ferriman-Gallwey score ≥8). (3) Polycystic ovarian morphology on ultrasound (ovaries reveal more than 10 cm³ of ovarian volume OR more than 12 follicles ranging in size from 2 to 9 mm). The sample size was calculated using the OpenEpi calculator with a confidence interval of 95%, power of 80% and an expected proportion of metabolic abnormality (insulin resistance to be 64%) among PCOS patients.^11^

Patients were divided into two groups: adolescents (aged 12-19 years) and adults (aged 20-40 years). Detailed clinical history was recorded, including menstrual irregularities, hirsutism, acne and weight changes. Physical examination assessed anthropometric measurements, such as body mass index (BMI), waist-to-hip ratio and blood pressure. Biochemical parameters, including serum levels of androgen (testosterone, dehydroepiandrosterone sulfate and androstenedione), HOMA-IR, lipid profile, fasting insulin and blood glucose levels were measured using standard laboratory techniques. Ultrasound examinations were performed to assess ovarian morphology. Prolactin, luteinizing hormone (LH) and follicle-stimulating hormone (FSH) were investigated using immunoassay method on 3^rd^ day of cycle at 9.00 am. Insulin resistance was assessed with the help of the HOMA-IR formula:


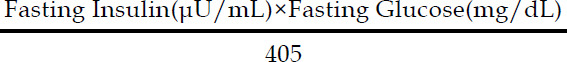


### Statistical Analysis:

Data were analyzed using SPSS version 26. Continuous variables were represented as mean±standard deviation, while categorical variables were represented as frequencies and percentages. For the comparison of continuous variables, Mann-Whitney U and independent t-tests were used, while chi-square tests were used for comparing categorical data. A p-value of less than 0.05 was considered as statistically significant.

## RESULTS

The mean age of PCOS patients was 24.5 ± 5.6 years, with a mean BMI of 27.8 ± 4.5 kg/m². Menstrual irregularities were present in 105 (70.0%) patients. Hirsutism, defined as a Ferriman-Gallwey (FG) score of ≥8, was observed in 92 (61.3%) patients, while acne was noted in 78 (52.0%). A favorable family history of PCOS was noted in 65 (43.3%) of cases. Rest of the findings are given in table (Table-I).

**Table 1:** Demographic Characteristics and Effect Modifiers of PCOS Patients (n = 150).

Characteristic	Mean ± SD / n (%)
Age (years)	24.5 ± 5.6
BMI (kg/m²)	27.8 ± 4.5
Waist-to-Hip Ratio	0.86 ± 0.08
Menstrual Irregularities	105 (70.0%)
Hirsutism (FG Score ≥ 8)	92 (61.3%)
Acne	78 (52.0%)
Family History of PCOS	65 (43.3%)
Family History of Diabetes	58 (38.7%)
Hypertension	22 (14.7%)

Mean fasting blood sugar level was 98.5 ± 12.3 mg/dL, with a mean fasting insulin concentration of 15.2 ± 6.4 μU/mL. The average HOMA-IR was 3.7 ± 1.6, indicating significant insulin resistance, with the average total level of testosterone of 62.4 ± 18.9 ng/dL. The average values of LH and FSH, Triglycerides, HDL and LDL are given in Table-II

**Table-II:** Biochemical Parameters of PCOS Patients (n = 150).

Parameter	Mean ± SD	Reference Range
Fasting Glucose (mg/dL)	98.5 ± 12.3	70-100
Fasting Insulin (μU/mL)	15.2 ± 6.4	<10
HOMA-IR	3.7 ± 1.6	<2.5
Total Testosterone (ng/dL)	62.4 ± 18.9	15-70
LH (mIU/mL)	11.2 ± 4.3	2-12
FSH (mIU/mL)	6.3 ± 2.1	3-10
Triglycerides (mg/dL)	145.8 ± 34.7	<150
HDL (mg/dL)	42.1 ± 8.5	>50
LDL (mg/dL)	126.3 ± 30.2	<130

Mean age of adolescent was 17 ± 1. Adolescents had a lower mean BMI (25.5 ± 3.8 kg/m²) when compared to adults (29.1 ± 4.9 kg/m², p < 0.001). Menstrual irregularities were more common in adolescents (68 [90.7%]) compared to adults (37 [49.3%], p < 0.001). Hirsutism was present in 48 (64.0%) adolescents and 44 (58.7%) adults with no significant difference (p = 0.52). The mean fasting insulin levels were significantly higher in adults (16.6 ± 6.5 μU/mL) than in adolescents (13.8 ± 5.9 μU/mL, p = 0.01). The mean HOMA-IR levels were significantly elevated in adults (4.1 ± 1.7) when compared to adolescents (3.2 ± 1.4, p = 0.002). The mean HDL levels were significantly higher in adolescents (45.3 ± 7.8 mg/dL) than in adults (39.2 ± 8.1 mg/dL, p < 0.001), while the mean LDL level was lower in adolescents (120.5 ± 28.6 mg/dL) when compared to adults (132.1 ± 31.4 mg/dL, p = 0.03). (Table-III).

**Table-III:** Comparison of clinical and biochemical features between adolescents and adults.

Parameter	Adolescents (n=75)	Adults (n=75)	p-value
BMI (kg/m²)	25.5 ± 3.8	29.1 ± 4.9	<0.001
Menstrual Irregularities	68 (90.7%)	37 (49.3%)	<0.001
Hirsutism (FG Score ≥ 8)	48 (64.0%)	44 (58.7%)	0.52
Fasting Insulin (μU/mL)	13.8 ± 5.9	16.6 ± 6.5	0.01
HOMA-IR	3.2 ± 1.4	4.1 ± 1.7	0.002
HDL (mg/dL)	45.3 ± 7.8	39.2 ± 8.1	<0.001
LDL (mg/dL)	120.5 ± 28.6	132.1 ± 31.4	0.03

Menstrual irregularities (78.8% vs. 58.5%, p = 0.01) and hirsutism (72.9% vs. 46.2%, p = 0.003) were more common in insulin-resistant patients, while fasting insulin (18.2 ± 4.8 vs. 9.8 ± 2.9 μU/mL, p < 0.001) and HOMA-IR levels (4.8 ± 1.2 vs. 2.3 ± 0.6, p < 0.001) were significantly higher in insulin-resistant patients. Total testosterone (69.2 ± 18.9 vs. 57.3 ± 15.6 ng/dL, p = 0.002) and LDL (133.3 ± 31.2 vs. 119.5 ± 28.1 mg/dL, p = 0.02) levels were elevated, while HDL was lower (40.1 ± 8.6 vs. 44.2 ± 7.5 mg/dL, p = 0.02) in insulin-resistant patients (Table-IV).

**Table-IV:** Stratification of outcome variables based on insulin resistance in PCOS patients.

Outcome Variable	IR Absent (n=65)	IR Present (n=85)	p-value
Menstrual Irregularities	38 (58.5%)	67 (78.8%)	0.01
Hirsutism (FG Score ≥ 8)	30 (46.2%)	62 (72.9%)	0.003
Fasting Insulin (μU/mL)	9.8 ± 2.9	18.2 ± 4.8	<0.001
HOMA-IR	2.3 ± 0.6	4.8 ± 1.2	<0.001
Total Testosterone (ng/dL)	57.3 ± 15.6	69.2 ± 18.9	0.002
HDL (mg/dL)	44.2 ± 7.5	40.1 ± 8.6	0.02
LDL (mg/dL)	119.5 ± 28.1	133.3 ± 31.2	0.02

## DISCUSSION

Polycystic ovary syndrome (PCOS) exhibits varying clinical and biochemical profiles in adolescents and adults, necessitating age-specific management strategies. Among the several metabolic dysfunctions that contribute to PCOS, insulin resistance plays a pivotal role, making it crucial to comprehend these differences to implement targeted interventions.^12^In our study we observed no significant difference in menstrual patterns between adolescent and adult PCOS patient consistent with the findings of Jain et al.^13^ Elevated testosterone levels were also in agreement with the findings of Jain et al.^13^ and Dahiya et al..^14^ In addition, our study showed that a significant proportion of patients presented with hyperandrogenemia without hyperinsulinemia, aligning with Jain et al. where 56% of adolescents and 60% of adults exhibited this pattern.^13^

In our study clinical parameters such as hirsutism (58% vs. 47%), weight gain (38% vs. 24%) and increased BMI (36% vs. 17%) were more prevalent in adults than adolescents, whereas oligomenorrhea (87% vs. 88%) and acne (44% vs. 46%) showed no significant difference between groups. These findings were in- agreement with the findings by Dahiya et al. (2020.^14^ Ahmed et al. (2020) reported that 49.13% of PCOS patients had moderate hypersecretion of LH, which was also evident in our study with a significant proportion of patients exhibiting elevated LH levels.^15^ Our study demonstrated that younger PCOS patients exhibited a significantly higher total testosterone levels, whereas fasting insulin, glucose levels, BMI and triglycerides were elevated in older age groups consistent with the findings of Liang et al. (2011).^16^ Furthermore, our results were in agreement with Dutt et al. (2022), who also found that gonadotropin levels did not significantly differ between adolescents and adults with PCOS.^17^

Our study observed a strong correlation between BMI and metabolic disturbances (including abnormal glucose levels and hyperinsulinemia) in overweight and obese patients, which corroborates the findings of Rajbanshi et al.^18^ Additionally, the correlation between menstrual cycle disturbances and BMI reported by Spandana et al. was also evident in our study, further highlighting the metabolic implications of PCOS.^19^ Lastly, Zhang et al. found significantly increased BMI, insulin resistance and LH levels in PCOS patients when compared to controls (p = 0.001). Our study corroborated these findings, with significantly higher LH, insulin resistance and testosterone levels in PCOS patients.^20^

Overall, our study was able to find remarkable difference between clinical and biochemical parameters of adolescent and adult PCOS. In Pakistan very limited data regarding this difference in parameter is available. This highlight the importance of the fact that PCOS should be studied in each and every age group from teen to post-menopausal women, so that diagnostic criteria can be modified age wise. This way doctors will be able to understand difference in treatment strategies for adolescent which is more symptomatic like improving quality of life and preventing long term health risk while adult prioritize fertility related issues and health related conditions like type 2 DM and cardiovascular risk factors. Various dietary and life style factors which enhance the severity and poor outcome of PCOS are junk food consumption, high sugar intake, low fibre diet, physical inactivity, sleep deprivation etc.^21^ People need to be educated about them so they can avoid them by adopting healthy life style. The significant overlap in trends across studies underscores the need for early diagnosis and tailored management strategies to mitigate the long-term consequences of PCOS.

### Limitations:

This study has some limitations, like it was single center cross section study and because of the financial constraint most of the patients, were unable to do advance investigations. Because of the increasing prevalence of PCOS across the different age group and keeping in mind the findings of our study we are planning to do multicenter studies with financial support, which will not only truly show the spectrum of this condition across different age group but will also help in overcoming these limitations.

## CONCLUSION

Our study confirms significant hormonal and metabolic abnormalities in both adolescents and adults with PCOS. The findings emphasize the need for routine metabolic screening across different age groups, early detection and targeted interventions of metabolic and reproductive complications related to PCOS. Comprehensive evaluation and timely treatment can improve long-term outcomes in affected individuals.

### Authors Contribution:

**BB:** Proposed the idea, designed the study, principal investigator, interpretation of data, reviewed the manuscript, statistical analysis, literature search, supervision, final approval.

**GF:** Principal investigator, designing the study, Data collection and compilation, Manuscript writing, statistical analysis, Literature search, Final approval, responsible for accuracy and integrity of work.

**MM:** Literature search, Principal investigator, Data collection and compilation, Manuscript writing, statistical analysis.

**HK:** Data collection, Literature search, Data interpretation, Drafting, Critical revision of article
